# Depressive symptoms in amyotrophic lateral sclerosis: associations with quality of life, disease progression and perceived social support—a German multicenter study

**DOI:** 10.1186/s40359-026-05273-z

**Published:** 2026-08-04

**Authors:** Nina Domke, Kristina Geue, Ina Schlichte, Markus Ullsperger, Thomas Meyer, Susanne Petri, Stefanie Schreiber, Stefan Vielhaber, Florian Junne, Susanne Vogt

**Affiliations:** 1 University Clinic of Psychosomatic Medicine and Psychotherapy, University Medicine, Otto-von-Guericke-University Magdeburg, Medical Faculty, Leipziger Straße 44, Magdeburg, D-39120 Germany; 2 University Clinic of Neurology, Otto-von-Guericke-University Magdeburg, University Hospital Magdeburg, Magdeburg, 39120 Germany; 3 Department of Neurology, Diakonissenanstalt, Flensburg, 24939 Germany; 4 Department of Neuropsychology, Otto-von-Guericke-University Magdeburg, Magdeburg, 39120 Germany; 5 Center for Behavioral Brain Sciences (CBBS), Magdeburg, 39106 Germany; 6 Center for ALS and Other Motor Neuron Disorders, Department of Neurology, Charité -Universitätsmedizin Berlin, Berlin, 13353 Germany; 7 Ambulanzpartner Soziotechnologie APST GmbH, Berlin, 13353 Germany; 8 Department of Neurology, Hannover Medical School, Hannover, 30625 Germany; 9 Department of Neurology, DIAKOVERE Henriettenstift, Hannover, 30625 Germany; 10 German Center for Neurodegenerative Diseases (DZNE), Magdeburg, 39120 Germany; 11 German Center for Mental Health (DZPG), Partner Site Halle-Jena-Magdeburg, Magdeburg, 39120 Germany

**Keywords:** Amyotrophic lateral sclerosis (ALS), Depressive symptoms, Health-related quality of life, Emotional well-being, Perceived social support, Disease progression

## Abstract

**Background:**

Amyotrophic Lateral Sclerosis (ALS) is a rapidly progressive, fatal neurodegenerative disease. Patients with ALS are at increased risk of developing depressive symptoms. A clearer understanding of the associations between depressive symptoms, disease progression, health-related quality of life and perceived social support is needed to advance intervention-oriented research and improve psychosocial care in ALS.

**Methods:**

In a multicenter cross-sectional study, patients with ALS were asked to complete patient-reported outcomes (PROs) to evaluate depressive symptoms (ALS-Depression-Inventory, ADI-12), health-related quality of life (ALS-Assessment-Questionnaire, ALSAQ-5/ALSAQ-40), physical functioning (ALS-Functional Rating Scale-Extension, ALSFRS-EX) and perceived social support (Multidimensional Scale of Perceived Social Support, MSPSS). To evaluate associations between demographic/clinical variables and PROs, we performed Pearson's, Spearman's, and partial correlation analyses. In addition, a multiple regression was run to identify predictors of depressive symptoms. Comparisons of patient groups were performed with chi-squared tests for categorical variables and Student t-tests or one-way analysis of variance (ANOVA) for continuous variables, followed by post-hoc tests.

**Results:**

Out of the 150 patients examined, 60% reported depressive symptoms, with half of them exhibiting clinically relevant depressive symptoms. Depressive symptoms were significantly associated with disease progression (r = .466, *p* < .001), various aspects of health-related quality of life (mobility: r = .394, activities of daily living: r = .268 and emotional well-being: r = .793, all *p* < .001), the MSPSS sum score (partial r = -.234, *p*  = .007), and the ALSFRS-EX sum score (r = -.352, *p* < .001). Patients with depressive symptoms reported significantly more physical impairments (*p* = .002) and less perceived social support by their families (*p* = .016) and friends (*p* < .001) compared to patients without depressive symptoms.

**Conclusions:**

Depressive symptoms are prevalent in patients with ALS and are closely associated with faster disease progression, reduced physical functioning, poorer health-related quality of life, and lower perceived social support. Further studies, particularly using qualitative methods, are needed to elucidate protective factors, clarify psychosocial mechanisms, and support the conceptualization of specialized and integrated psychosocial care for patients with ALS in Germany.

## Introduction

Amyotrophic lateral sclerosis (ALS) is a fast progressive and incurable degenerative motor neuron disease. The mean age of onset is 66 years and in most cases the disease leads to death due to respiratory failure within 3–5 years [1]. Treatment is mainly symptomatic and should be carried out by a multidisciplinary team of specialists aiming to maintain health-related quality of life (hrQoL) [2]. People living with ALS often experience psychological distress. A longitudinal study investigating anxiety and depression in people with ALS showed that almost half of patients exhibit symptoms of anxiety soon after diagnosis, although the prevalence decreased within the first six months following the diagnosis [3]. A systematic review compiling results of studies investigating anxiety and depression in people living with ALS shows that patients often experience symptoms of both disorders [4]. These findings are further supported by a meta-analysis reporting a pooled prevalence of depressive symptoms of 34% in patients with ALS, reaching up to 50% depending on the assessment instrument used to evaluate the depressive symptoms [5].

Previous studies on the correlation between depressive symptoms and disease progression showed either no associations or significant associations with a small effect size [6–8]. Evidence concerning the association between depressive symptoms/depression and hrQoL has also been inconsistent, studies reporting both a negative association [9, 10] as well as an absence of significant associations [11]. Several studies have demonstrated a positive relationship between social support and quality of life in patients with ALS [2, 12–15]; however, few studies have investigated the relationship between perceived social support and depressive symptoms in patients with ALS [16–18]. A German longitudinal study on psychological adjustment in patients with ALS provided initial evidence that higher levels of perceived social support, assessed by the Berlin Social Support Scales, were predictive of lower levels of depressive symptoms and higher individual quality of life [18]. However, the study was performed on a small sample of 27 patients, which limits the conclusiveness of its findings. Further research on the different dimensions of perceived social support in relation to depressive symptoms and physical impairments in ALS is needed to better understand this psychosocially relevant issue from the patients’ perspective. According to a systematic review on patients with mental illness, there is clear evidence for an association between poorer perceived social support and depressive symptoms [19]. These findings are supported by a current systematic review on patients with chronic pain, in which 15 out of 16 studies reported a negative association between depression and perceived social support [20].

In conclusion, depressive symptoms are prevalent among patients with ALS and contribute an additional strain beyond the multifaceted somatic symptomatology. Further investigation is needed to evaluate how depressive symptoms in ALS are associated with disease progression, perceived social support and hrQoL. The consistent use of ALS-specifically validated patient-reported outcomes (PROs) may help develop a deeper understanding of patient experiences, facilitating intervention-oriented research and improving psychosocial care for patients with ALS.

The association between depressive symptoms and disease progression, hrQoL, and perceived social support, has not yet been clarified in patients with ALS. Therefore, this study aimed to investigate how depressive symptoms are associated with disease progression, physical impairments, multiple dimensions of hrQoL, and different sources of perceived social support.

## Methods

### Study design

The study was approved by the research ethics committee of the Medical Faculty of the University of Magdeburg (Ethic Votum 38/15) and was part of a previous multi-center cross-sectional study in patients with ALS [21, 22]. Between June 2015 and June 2019, patients aged 18 years or older, who had been diagnosed with ALS according to the revised El Escorial criteria [23] participated in the study after providing written informed consent. The patients were recruited from the specialized ALS outpatient clinics at the university hospitals in Magdeburg, Hannover and Berlin. Exclusion criteria were insufficient German language skills that would hinder understanding of the questionnaires, or severe cognitive impairments, as assessed by clinical judgment during the neurological consultation. Demographic and clinical data, such as the region of onset and disease duration, were recorded during a scheduled telephone call with the patients. In cases of pronounced bulbar symptoms or dyspnea-induced inability, interviews were conducted with the assistance of a family member. The patient-reported assessment instruments were sent home by mail. If a patient was unable to complete the forms independently due to pronounced physical limitations, a family member or caregiver was authorized to assist according to the patient’s instructions. The completed forms were then returned using prepaid envelopes.

### Patient characteristics

The patients were categorized based on the onset of symptoms into bulbar, upper or lower limb onset [24]. Clinical impairment was further classified using the King’s clinical staging system for ALS, which is based on disease milestones derived from the ALS Functional Rating Scale [25, 26].

### Measures

All survey instruments are patient-reported outcome measures and have been validated in the German language.

#### Depressive symptoms

The ALS-Depression Inventory (ADI-12) is a disease-specific screening instrument which includes 12 items. The items can be answered on a 4-point Likert scale ranging from 1 (“I fully agree”) to 4 (“I do not agree at all”). A sum score above 28 points is classified as clinically relevant depressive symptoms and between 22 and 28 points as mild depressive symptoms. A total score below 22 points indicates the absence of depressive symptoms [27, 28].

According to the developers of the original German version, the ADI-12 measures a construct described as “mood, anhedonia and energy” and is intended to yield a single total score [28]. For exploratory purposes, a complementary analysis of potential differences in the association pattern of depressive symptom clusters with social support [29, 30] and disease progression was performed. Therefore, the items of the ADI-12 were grouped into a two- factor structure of anhedonia and negative mood/lack of energy proposed in an Italian psychometric study of the ADI-12 [31]. However, this two-factor structure is yet to be formally replicated in the German version of the ADI-12.

#### Perceived social support

This construct is evaluated by the Multidimensional Scale of Perceived Social Support (MSPSS) which distinguishes the context of the respective perceived social support in three subscales (family, friends and significant others) [32]. The scale comprises 12 items which are rated on a 7-point Likert scale ranging from 1 (“very strongly disagree”) to 7 (“very strongly agree”). The sum score can range from 12 to 84 points, and the subscale scores from 4 to 28. A higher score indicates a higher level of perceived social support.

#### Health-related quality of life (hrQoL)

Health-related quality of life is assessed using the ALS-Assessment Questionnaire (ALSAQ). The questionnaire is available in a long form (ALSAQ-40) and in a short form (ALSAQ-5) version [33, 34]. The ALSAQ-40 contains 5 domains: physical mobility (10 items), activity of daily living/independency (10 items), eating and drinking (3 items), communication (7 items), and emotional functioning (10 items). The ALSAQ-5 contains one item for each subscale. For this study, the ALSAQ-5 was used for the subscales “physical mobility”, “activities of daily living and independence”, “eating and drinking”, and “communication”. For the subscale “emotional functioning”, the ALSAQ-40 with 10 items for the respective scale, was used to assess the different facets of emotional well-being in more detail. The respective subscale raw score was transformed into a 0–100 range with higher scores indicating more impairments in hrQoL.

#### Physical functional impairments

The functional status of the patients was rated with the ALS-Functional Rating Scale which is the most widely used functional rating scale in clinical practice as well as in ALS research. It shows excellent reliability in terms of self- vs evaluator administration as well as online assessment compared to in-clinic evaluation [35–37]. However, there is evidence that patients and caregivers using the self-administered form tend to rate themselves or the patients on average one point higher (better function) than clinicians [37]. Several different versions have been created over the past 25 years [38] and a recent review provides an in-depth analysis of various remote assessment versions of the ALSFRS-R [39]. In our study we used the ALS-Functional Rating Scale-Extension (ALSFRS-EX) which was validated as a patient-reported outcome in German [40, 41]. The ALSFRS-EX consists of four subscales (“bulbar”, “fine motor”, “gross motor”, “respiratory”) and comprises 15 items. The sum score ranges from 0 to 60 points with a lower sum score indicating a worse condition of physical functioning.

The ALSFRS-EX was used to estimate disease progression using the following formula: (60-sum of ALSFRS-EX)/disease duration from symptom onset to investigation date in months. This approach is described by Ellis et al. [42], but was adapted by using the ALSFRS-EX scale, which has been validated in German [40].

### Statistical analysis

Statistics were analyzed with IBM SPSS, Version 29.0 (IBM Corporation, Armonk, New York, USA). Demographic and clinical characteristics of the participants were described using frequencies (%) and means with standard deviations. Bivariate correlations were conducted between demographic and clinical data, ADI-12, ALSFRS-EX, ALSAQ and MSPSS, using the Pearson or Spearman coefficient with 95% confidence intervals. Eta-Coefficient was used to determine correlations between nominal and interval variables. As preliminary analyses of our dataset revealed a significant correlation with a large effect size between disease progression and depressive symptoms, we conducted partial correlation analyses to control for a potential confounding effect of disease progression. A nonparametric bootstrap with 1,000 resamples was used to calculate 95% confidence intervals. Correlations were interpreted as “very small” (r = 0.05), „small “ (r = 0.10), „medium “ (r = 0.20), „large “ (r = 0.30) und „very large “ (r = 0.40) [43]. For the interpretation of Cohen’s d values, effect sizes between 0.10 and 0.29 were considered weak, from 0.30 to 0.49 moderate, and from 0.50 to 1.00 strong [44]. A multiple regression analysis was performed to identify predictors of the ADI-12 sum score, using all parameters that significantly correlated with the score. Comparisons of patient groups were performed with chi-squared tests for categorical variables and Student t-tests or one-way analysis of variance (ANOVA) for continuous variables, followed by post-hoc tests. Unless stated otherwise, significance level was set at α ≤ 0.05. Bonferroni adjustments were used if necessary.

## Results

### Demographic and clinical data

A total of 182 patients were initially recruited for the study. Of these, 150 patients with ALS were included in the study and 147 patients completed all questionnaires (ADI-12, MSPSS, ALSAQ-5, and ALSAQ-40 subscale “emotional functioning”). A detailed flow diagram of recruitment, including dropouts, is provided elsewhere [22]. Eighty-nine of the 147 patients (60% of the patients) had depressive symptoms (ADI-12 score ≥ 22 points), of whom 45 suffered from clinically relevant depressive symptoms (ADI-12 score > 28 points). The demographic and clinical data, along with the patient-reported outcomes for patients with and without depressive symptoms, are presented in Table 1. Patients with depressive symptoms had a significantly lower ALSFRS-EX sum score than patients without depressive symptoms. On the ALSFRS-EX subscore level, patients with depressive symptoms had significantly lower ALSFRS-EX subscores for “fine motor” (*p* = 0.007), “gross motor” (*p* = 0.002) and “respiratory” (*p* = 0.002) function. Perceived social support through “family” and “friends” was significantly higher in patients without depressive symptoms (family *p* = 0.016,friends *p* < 0.001). The ALSAQ-5 subscale scores “mobility” and “activities of daily living” as well as the ALSAQ-40 subscale score “emotional functioning” were significantly higher in patients with depressive symptoms (*p* < 0.001).

**Table 1:** Demographic and clinical data as well as results of patient-reported outcomes, differed in patients with and without depressive symptoms (mean ± SD or number of patients (%))

	Patients with depressive symptoms, ***n*** = 89	Patients without depressive symptoms, ***n ***= 58	***p***-value	Cohen’s d
Gender (female/male)	30/59 (33.7/66.3)	13/45 (22.4/77.6%)	.098	
Patient age in years	61.5 ± 15.3	58.5 ± 16.2	.133	0.192
Disease duration from diagnosis in months	35.1 ± 49.0	36.9 ± 37.6	.406	0.040
Symptom onset			.803	
Bulbar	18 (20.2%)	14 (24.1%)		
Upper limb	35 (39.3%)	22 (37.9%)		
Lower limb	33 (37.1%)	19 (32.8%)		
King’s clinical staging			**.013**	
Stage 1: Symptom onset/functional involvement of first region				
Stage 2A: Diagnosis	4 (4.5%)	8 (13.8%)		
Stage 2B: Functional involvement of second region	9 (10.1%)	14 (24.1%)		
Stage 3: Functional involvement of third region	39 (43.8%)	21 (36.2%)		
Stage 4A: Need for gastrostomy	6 (6.7%)	5 (8.6%)		
Stage 4B: Need for respiratory support (NIV)	31 (34.8%)	10 (17.2%)		
ADI-12				
Sum score	29.1 ± 5.7	17.1 ± 2.9	** <.001**	**2.501**
Mild depressive symptoms (22–28 points)	24.4 ± 1.9 (n = 44, 29.3%)			
Clinically relevant depressive symptoms (> 28 points)	33.7 ± 4.2 (n = 45, 30%)			
ALSFRS-EX				
Sum score	36.6 ± 11.7	43.2 ± 12.4	**.002**	**0.551**
Bulbar subscore	11.6 ± 4.2	12.3 ± 3.8	.162	0.173
Fine motor subscore	7.9 ± 4.5	9.9 ± 4.8	.**007**	0.433
Gross motor subscore	8.1 ± 4.5	10.6 ± 5.1	.**002**	0.527
Respiratory subscore	9.0 ± 3.3	10.5 ± 2.5	.**002**	0.498
ALSAQ				
ALSAQ-5 subscale score “mobility”	2.1 ± 1.4	1.1 ± 1.5	** <.001**	**0.694**
ALSAQ-5 subscale score “activities of daily living”	2.3 ± 1.4	1.5 ± 1.4	** <.001**	**0.571**
ALSAQ-5 subscale score “eating/drinking”	1.3 ± 1.5	.95 ± 1.5	.102	0.233
ALSAQ-5 subscale score “communication”	1.9 ± 1.5	1.6 ± 1.5	.077	0.200
ALSAQ-40 subscale score “emotional functioning”	48.1 ± 19.7	19.7 ± 13.1	** <.001**	**1.632**
MSPSS				
Sum score	69.0 ± 11.4	74.4 ± 9.3	**.002**	**0.508**
Family subscore	24.0 ± 4.0	25.4 ± 3.6	**.016**	**0.364**
Friends subscore	20.5 ± 6.0	23.9 ± 3.9	** <.001**	**0.645**
Significant other subscore	25.0 ± 3.8	25.1 ± 4.9	.861	0.023
Disease progression	1.00 ±.94	.68 ± 2.1	.287	0.212

Significant *p*-values are boldfaced

Bonferroni-adjusted *p*-values for the subscales of ALSFRS-EX:.05/4 =.0125; ALSAQ: .05/5 =.01; MSPSS:.05/3 =.016

*ADI-12* ALS-Depression Inventory, *ALSFRS-EX* ALS Functioning Rating Scale – Extension, *ALSAQ* ALS Assessment Questionnaire, *MSPSS* Multidimensional Scale of Perceived Social Support

### Depressive symptoms and disease progression

**Fig. 1:**
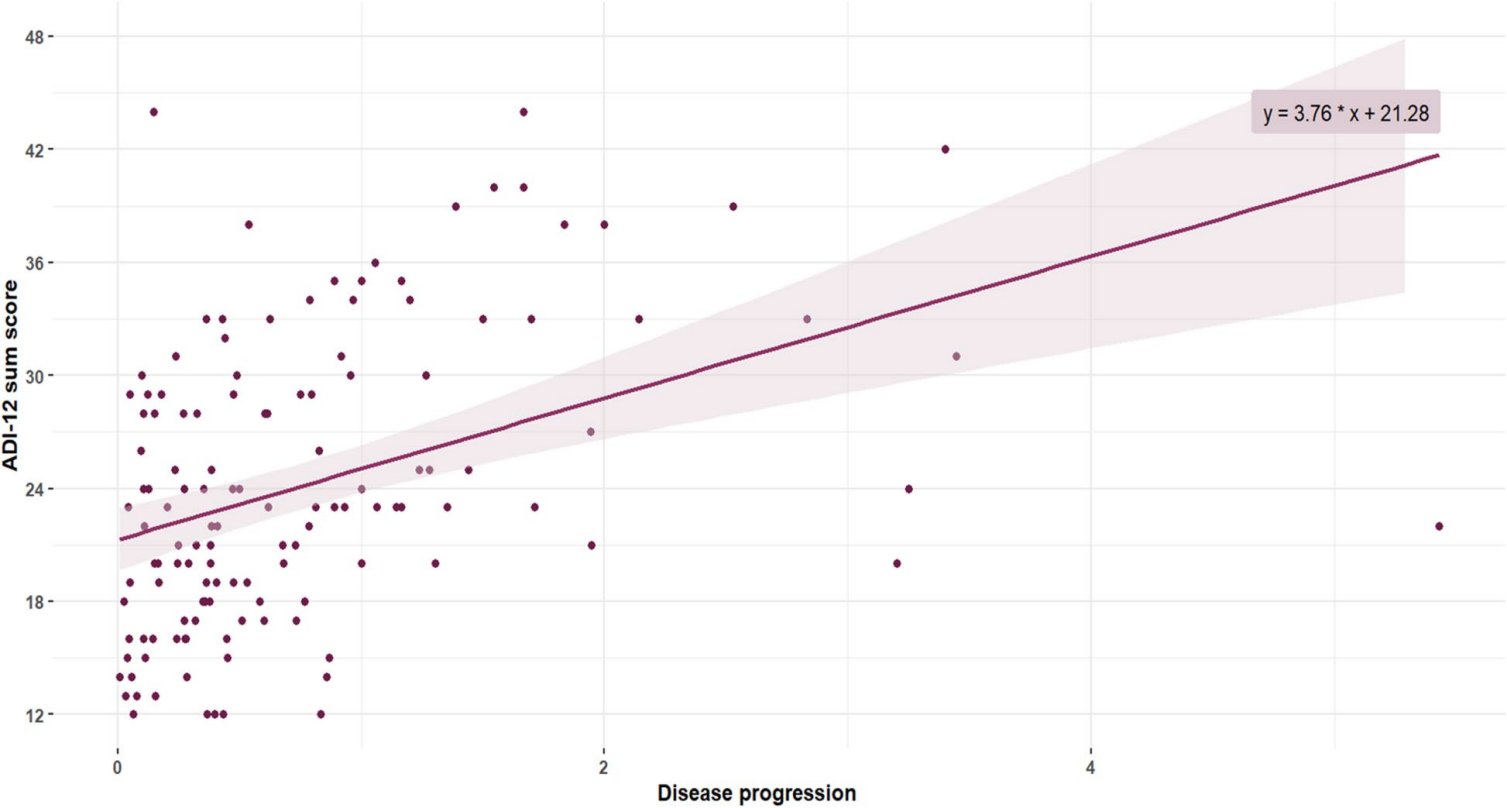
Relationship between depressive symptoms (sum score of the ALS-Depression Inventory - ADI-12) and disease progression. A linear regression line has been superimposed

The disease progression proved to be significantly correlated to the ADI-12 sum score with a large effect size (r = 0.466, *p* < 0.001; see Fig. 1). Further exploratory analysis of the depressive symptom subfactors anhedonia and negative mood/lack of energy also revealed significant correlations with large and very large effect sizes (anhedonia r = 0.384, *p* < 0.001 and negative mood/lack of energy 0.463, *p* < 0.001), respectively.

### Depressive symptoms, health-related quality of life and physical functional impairments

**Fig. 2:**
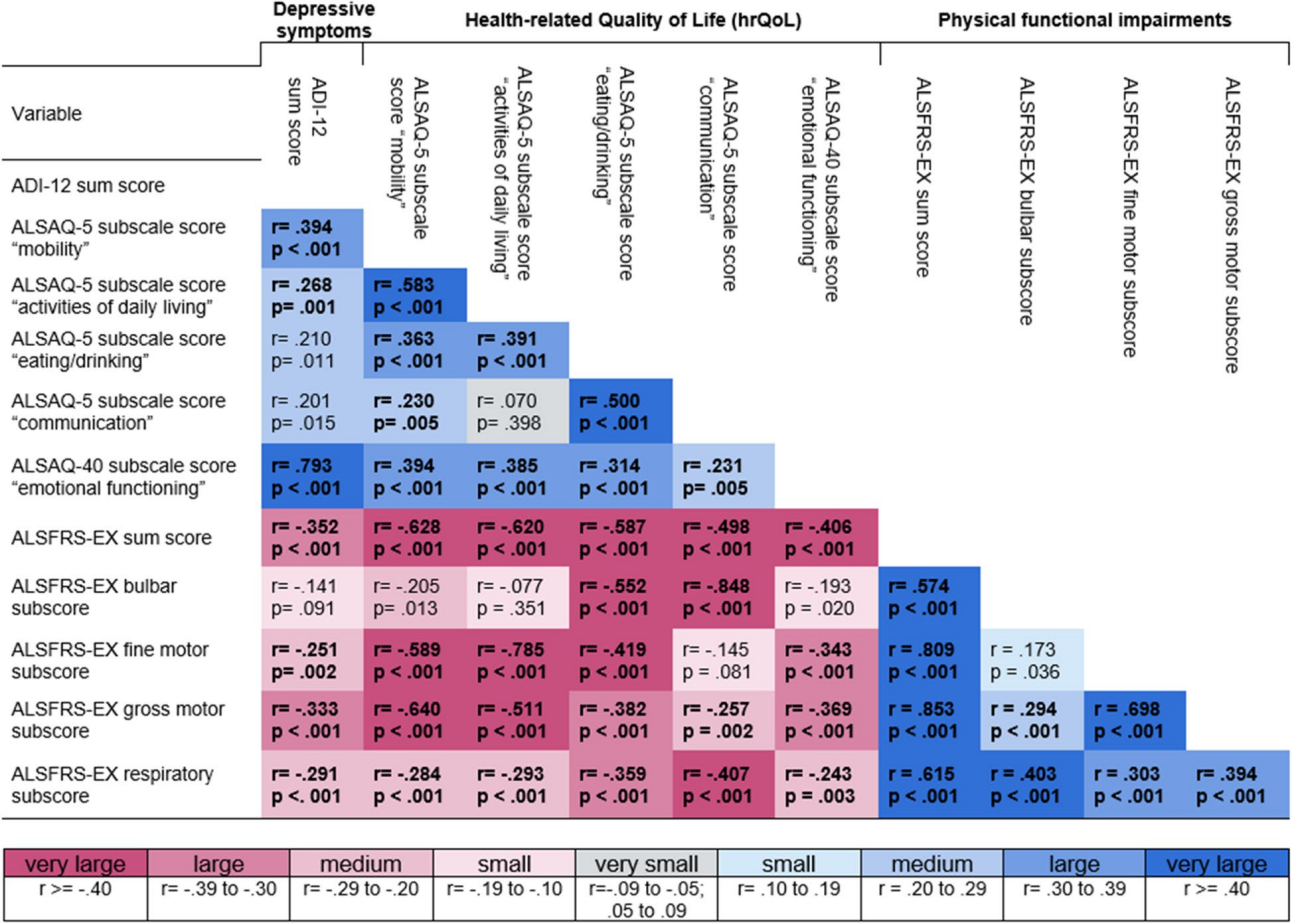
Correlation matrix with the correlations between depressive symptoms, health-related quality of life and physical functioning. A color gradient is used for visualization, with red indicating a negative correlation and blue a positive correlation. Color intensity reflects the magnitude of the correlation based on effect size interpretation according to Funder & Ozer [43]: very small (.05—–.09), small (.10—–.19), medium (.20—–.29), large (.30—–.39) and very large (≥> =.40). For subscales of instruments, appropriate Bonferroni-adjustments were made (ALSFRS-EX:.05/4 =.0125,ALSAQ:.05/5 =.01). Significant p-values are boldfaced

The results of the bivariate correlations between the ADI-12 sum score and the ALSAQ-5/ALSAQ-40 subscale scores as well as the ALSFRS-EX sum score and subscores are shown in the correlation matrix in Fig. 2. Significant correlations were observed between the ADI-12 sum score and the ALSAQ-40 subscale score “emotional functioning” (r = 0.793, 95% CI [0.793, 0.854], *p* < 0.001), as well as the ALSAQ-5 subscale scores “mobility” (r = 0.394, 95% CI [0.247, 0.531], *p* < 0.001) and “activities of daily living” (r = 0.268, 95% CI [0.112, 0.422], *p* = 0.001).

### Depressive symptoms, perceived social support and physical functioning

**Fig. 3:**
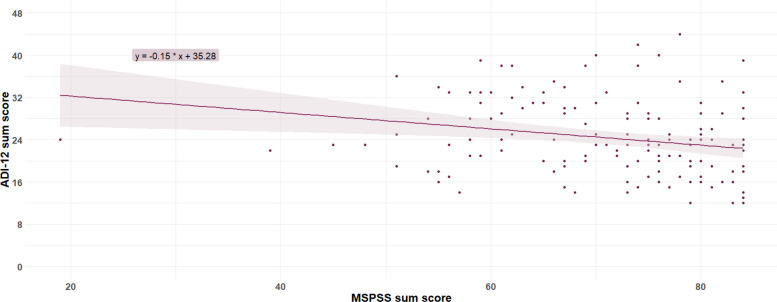
Relationship between depressive symptoms (sum score of the ALS-Depression Inventory—ADI-12) and perceived social support (sum score of the Multidimensional Scale of Perceived Social Support—MSPSS). A linear regression line has been superimposed

The ADI-12 and MSPSS sum scores were inversely related (Fig. 3). A higher level of perceived social support was associated with a lower level of depressive symptoms.

Referring to the partial correlation analysis between the ADI-12 sum score and the MSPSS sum score, a significant negative correlation with a medium effect size was observed (partial r = −0.234, 95% CI [−0.383, −0.101] *p* = 0.007) independent of disease progression. Further analysis for negative mood/lack of energy and the MSPSS sum score revealed a significant negative correlation with a medium effect size (partial r = −0.247, 95% CI [−0.390, −0.127], *p* = 0.004), while for anhedonia the correlation with the MSPSS sum score was not significant (partial r = −0.160, 95% CI [−0.317, 0.034], *p* = 0.066). Partial correlation analyses of the ADI-12 sum score with the MSPSS subscale scores (Bonferroni-adjustment 0.05/3 = 0.016) showed significant correlations for “friends” and “family” indicating a lower perceived social support in higher levels of depressive symptoms independent of disease progression. The correlation with the MSPSS subscale “friends” (partial r = −0.326, 95% CI [−0.465, −0.169] *p* < 0.001) was large and with the MSPSS subscale “family” (partial r = −0.174, 95% CI [−0.314, −0.050] *p* = 0.047) small. No significant correlation was observed with the MSPSS subscale “significant others” (partial r = −0.016, 95% CI [−0.149, −0.080] *p* = 0.858). In addition, a multiple regression with all parameters that significantly correlated with the ADI-12 sum score (MSPSS subscales “family” and “friends”, ALSFRS-EX, disease progression, gender) was run. The results of the regression showed that the model explained 17.6% of the variance (adjusted R^2^ = 0.176, *p* < 0.001). The MSPSS subscale “friends” significantly predicted depressive symptoms (ß = −0.360, *p* = 0.005), as did the ALSFRS-EX sum score (ß = −0.125, *p* = 0.019). The other variables did not.

**Fig. 4:**
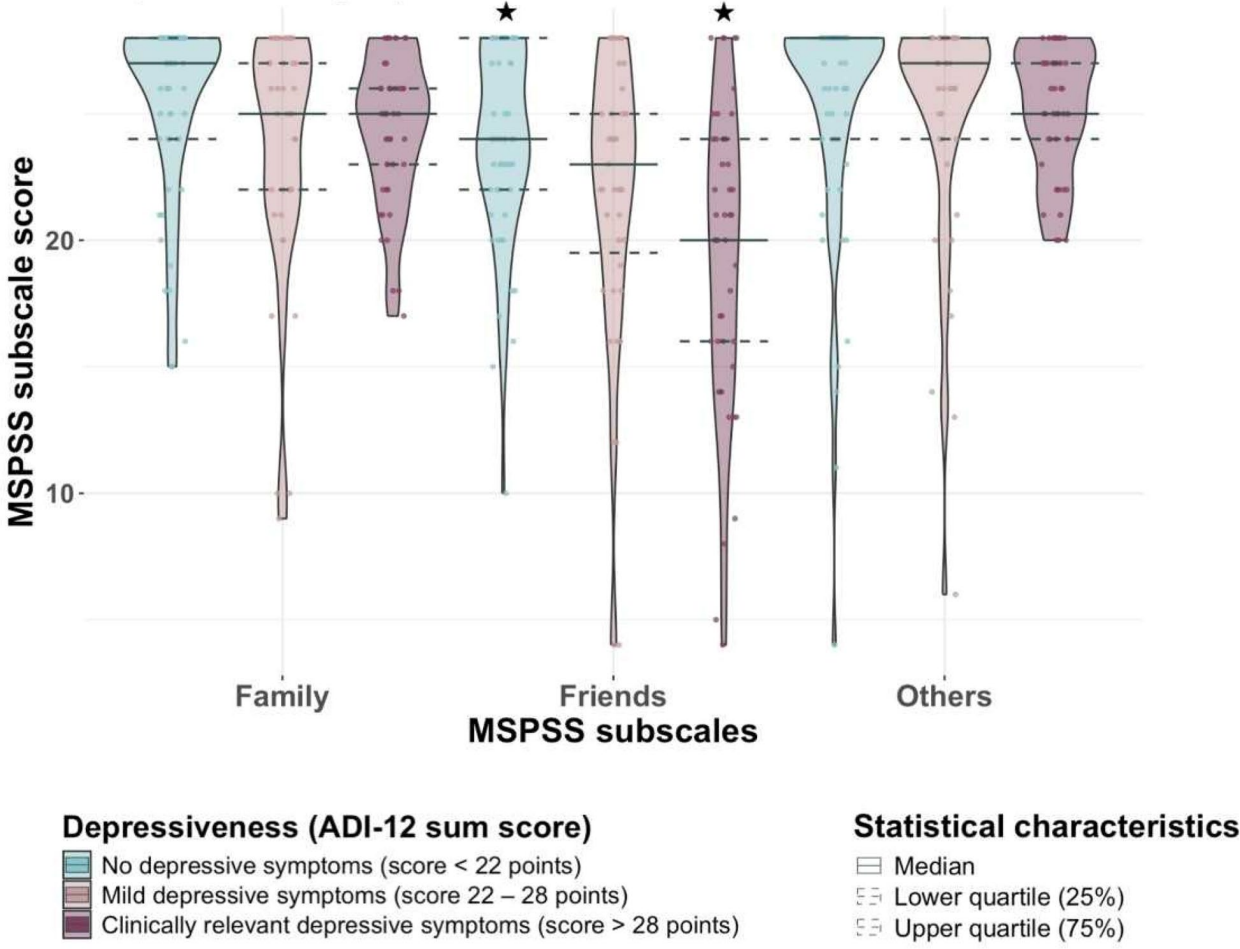
Perceived social support in relationship to depressive symptoms. Violin plots presenting the score distribution of the Multidimensional Scale of Perceived Social Support (MSPSS) subscales “family”, “friends” and “others” in relation to the levels of depressive symptoms according to the ALS-Depression Inventory (ADI-12) “no depressive symptoms”, “mild depressive symptoms”, “clinically relevant depressive symptoms”). Significant group differences in the subscale “friends” between the severity level “no depressive symptoms” and “clinically relevant depressive symptoms” (p = <.001) are highlighted by boldfaced stars

The MSPSS subscale scores in relation to the levels of depressive symptoms are presented in Fig. 4. A one-way ANOVA revealed that there was a statistically significant difference in the mean MSPSS scores of the subscale “friends” between the levels of depressive symptoms. Bonferroni’s test for multiple comparisons found that the significant difference existed between the severity level “no depressive symptoms” and “clinically relevant depressive symptoms” (p = < 0.001). There were no significant differences within the MSPSS subscales “family” (*p* = 0.068) and “significant others” (*p* = 0.942).

The sum score of the ALSFRS-EX significantly correlated with the MSPSS sum score with a medium effect size (r = 0.219, 95% CI [0.043, 0.371] *p* = 0.008) and with the ADI-12 sum score with a large effect size (r = 0.352, 95% CI [−0.512, −0.169] *p* < 0.001).

## Discussion

This study demonstrates that depressive symptoms were prevalent in patients with ALS and were significantly associated with the progression of the disease and various aspects of hrQoL. Correlation analyses revealed significant correlations between depressive symptoms and physical functioning, disease progression and perceived social support. Higher levels of depressive symptoms were significantly associated with lower perceived social support from friends and family, regardless of disease progression. Furthermore, regression analyses suggested that depressive symptoms and perceived social support were associated with physical functioning, but not with disease progression or gender. Patients with depressive symptoms reported significantly more impairments of physical functioning and felt significantly less supported by their family and friends compared to those without depressive symptoms.

### Depressive symptoms and disease progression

In our study cohort, 60% of the patients reported depressive symptoms, with one third reporting clinically relevant depressive symptoms according to the ADI-12, which highlights the substantial psychological burden in this population. The prevalence observed in our cohort is higher than that reported in a pooled analysis from a systematic review and meta-analysis [5], further underscoring this burden. Our findings are consistent with a previous study on German patients, which also used the ALS-specific ADI-12 to evaluate depressive symptoms [45].

Our data demonstrates a large correlation between depressive symptoms and the progression of the disease. A previous study revealed an association between depressive symptoms and disease progression with a small effect size [7]. In this study, disease progression was only correlated with a single item of the ALSAQ-40 subscale “emotional functioning” (“I have felt depressed”), and no specific measure of depressiveness was used. Another study rather reported a tendency towards a faster disease progression in patients with ALS and comorbid depression [6]. It is reasonable to assume that patients with ALS and depressive symptoms may have a lower treatment motivation and adherence to therapy, which may promote a faster progression of the disease, and, in turn, a faster progression may worsen the depressive symptoms. However, this remains speculative and in clinical practice, patients with ALS suffering from depressive symptoms need to be considered in their individual context. Research, particularly qualitative studies from both patient and caregiver perspectives, is needed to explore how accelerated disease progression may contribute to depressive symptoms and, conversely, how depressive symptoms may be linked to faster deterioration of the condition. Additional analyses are warranted to examine mortality among patients with ALS in relation to depressive symptoms and relevant clinical parameters, as well as in the context of psychotherapeutic and/or pharmacological interventions.

### Depressive symptoms, health-related quality of life and physical functional impairments

This study demonstrated associations between depressive symptoms and physical impairments as well as health-related quality of life in terms of mobility, activities of daily living, and emotional well-being. In our cohort, patients with depressive symptoms reported significantly poorer physical functioning. Similar findings were reported in an Italian study, which showed that both diagnostic phase and motor functional status significantly impact mood of patients with motor neuron diseases [46]. The observed association between physical functioning and depressive symptoms may be influenced by either pre-existing group differences in physical impairments or by a heightened perception of physical limitations among patients with depressive symptoms. The causal direction of this relationship remains unclear, and further longitudinal studies are needed to clarify these effects.

Several studies confirm the correlation between hrQoL and depressive symptoms [2, 10, 45, 47–49]; however, findings regarding the association between depressive symptoms and physical impairments have been inconclusive [45, 47].

As there is an overlap between the scales used to assess depressive symptoms and quality of life-related emotional well-being, our findings of a significant correlation with a large effect size between depressive symptoms and emotional well-being are unsurprising. For instance, the ALSAQ-40 subscale “emotional functioning” includes items which assess hopelessness and depressiveness (item 34: “I have felt hopeless about the future” and item 38 “I have felt depressed”), which corresponds to aspects also measured by the ADI-12.

### Depressive symptoms, perceived social support and physical functioning

The construct of social support is multidimensional and commonly a distinction between received social support and perceived social support is made [50]. The latter describes a person's beliefs on the quality and amount of social support they receive, which may be available through social contacts and relationships. Received social support, on the other hand, refers to the quantity of supportive behaviors an individual receives [19]. In a British study which used the MSPSS to investigate perceived social support in patients with motoneuron disease, there was a significant negative correlation between social support and depression, suggesting patients with higher levels of social support had lower levels of depression [16]. The level of perceived social support in our study cohort is comparable to that of the latter study. We also found that patients with depressive symptoms had significantly lower perceived social support compared to those without depressive symptoms, and that depressive symptoms were significantly associated with lower perceived social support. The latter finding is in line with a German study on patients with ALS, where higher social support significantly predicted lower depressive symptoms [17]. In our study, perceived social support was negatively associated with negative mood/lack of energy rather than with anhedonia. This observation aligns with previous literature suggesting that anhedonia acts as a barrier to engagement, motivation and enjoyment of social contexts [29].

The results of our regression analysis further suggest that depressive symptoms are significantly associated with perceived social support through friends and physical functioning, but not with disease progression or gender. The importance of social support for patients with ALS has been emphasized in a qualitative interview study where patients with ALS had a clear desire to participate in social activities and maintain social relationships. Patients considered technical aids for mobility and communication to be very important for this purpose [51]. In order to meet patients’ needs, we recommend providing information on additional sources of social support beyond their family and friends, and promoting social support and participation through concrete measures, such as psychosocial services and the timely provision of assistive devices. Coordinated multidisciplinary care has been shown to positively influence hospitalization rates and the clinical course of ALS [52]. Integrating psychosocial support within multidisciplinary care pathways that considers the stage and severity of the disease and addresses depressive symptoms may further contribute to improve patients’ overall wellbeing.

## Limitations

This study contains several limitations. First, the underlying study cohort was recruited from university-based outpatient clinics. It is possible, that these patients have a higher adaptive coping ability, which can influence depressiveness. Second, no formal assessment of cognitive and behavioral functioning or anxiety was conducted in the study. Furthermore, this study investigated depressive symptoms rather than referring to the clinical diagnosis of depression and refers to a slightly older data set from before the COVID-19 pandemic. In addition, the ADI-12 was used as a disease-specific screening instrument that yields a global score and does not include formally established subscales. Although an Italian psychometric study hints at a two-factor structure, namely “Negative Mood and Lack of Energy” and “Anhedonia” [31], this structure has not yet been replicated in Germany. Therefore, analyses of these subfactors were exploratory in nature and should be interpreted with caution. Future studies should consider combining global screening measures with multidimensional diagnostic tools. A potential limitation could also be that the ALSFRS-EX which was validated in German as a patient-reported outcome measure, has no formally established interrater reliability between patient and caregiver at home. Furthermore, it remains unclear who actually completed the questionnaires used in this study and whether the proxies provided the patient perspective that was required. Moreover, although disease duration from diagnosis was similar in patients with and without depressive symptoms, those with depressive symptoms had a lower self-reported physical functional status (ALSFRS-EX sum score) and a higher derived disease severity according to the King’s clinical staging system, which may have influenced the results of our study. Along with the cross-sectional design and the use of self-reported measures, these limitations restrict causal interpretation of the observed association between depressive symptoms and physical impairments.

## Conclusion

Our study suggests that depressive symptoms are prevalent in patients with ALS and are negatively associated with the progression of the disease and various dimensions of health-related quality of life. There are significant correlations between perceived social support, depressive symptoms, and physical impairment. The results of our study contribute to a better understanding of the associations between psychosocial and somatopsychic aspects in depressive ALS patients. In Germany, there is an unmet need for low-threshold psychosocial care for patients with ALS [53, 54], with 36% of patients looking for psychosocial support on the internet [55]. However, the provision of such care faces numerous barriers and challenges, particularly given the severity and immobilizing nature of the disease and often limited financial ressources, even in specialized centers [54]. It is especially challenging in rural areas where, according to a study by the Bundespsychotherapeutenkammer (German Federal Chamber of Psychotherapists), waiting times are significantly longer than in larger cities due to a scarcity of human ressources [56]. Further research is needed to elucidate the needs and preferences of patients with ALS, in order to develop specialized and coordinated psychosocial care, which addresses depressive symptoms and may help to maintain or enhance quality of life. While comparable psychosocial care programs exist for other patient groups with severe somatic illnesses, such as in psycho-oncology, ALS has received notably less attention, partly reflecting its rarity and the associated structural and research-related challenges. Notably, despite the severe physical and palliative burden of this devastating disease, around 40% of the patients did not exhibit depressive symptoms, which may indicate psychological resilience in this subgroup of patients. Identifying protective factors, such as coping strategies and perceived social support, is highly relevant for the development of targeted psychosocial interventions. Future research should focus on these resilience mechanisms to better understand and address depressive symptoms and enhance quality of life for patients with ALS.

## Data Availability

All data supporting the findings of this study are available within the paper.

## Abbreviations

ALS: Amyotrophic Lateral Sclerosis (ALS)
PRO: Patient-reported outcome
ADI-12: ALS-Depression-Inventory
ALSAQ: Amyotrophic Lateral Sclerosis Assessment Questionnaire
ALSFRS-EX: ALS-Functional Rating Scale-Extension
MSPSS: Multidimensional Scale of Perceived Social Support
ANOVA: One-way analysis of variance
hrQoL: Health-related quality of life
